# Employing plant functional groups to advance seed dispersal ecology and conservation

**DOI:** 10.1093/aobpla/plz006

**Published:** 2019-02-07

**Authors:** Clare Aslan, Noelle G Beckman, Haldre S Rogers, Judie Bronstein, Damaris Zurell, Florian Hartig, Katriona Shea, Liba Pejchar, Mike Neubert, John Poulsen, Janneke HilleRisLambers, Maria Miriti, Bette Loiselle, Edu Effiom, Jenny Zambrano, Geno Schupp, Gesine Pufal, Jeremy Johnson, James M Bullock, Jedediah Brodie, Emilio Bruna, Robert Stephen Cantrell, Robin Decker, Evan Fricke, Katie Gurski, Alan Hastings, Oleg Kogan, Onja Razafindratsima, Manette Sandor, Sebastian Schreiber, Rebecca Snell, Christopher Strickland, Ying Zhou

**Affiliations:** 1 Landscape Conservation Initiative, Northern Arizona University, Flagstaff, AZ, USA; 2 Department of Biology, Utah State University, Logan, UT, USA; 3 Department of Ecology, Evolution, and Organismal Biology, Iowa State University, Ames, IA, USA; 4 Department of Ecology and Evolutionary Biology, University of Arizona, Tucson, AZ, USA; 5 Dynamic Macroecology, Landscape Dynamics, Swiss Federal Research Institute WSL, Zürcherstrasse, Birmensdorf, Switzerland; 6 Faculty of Biology and Pre-Clinical Medicine, University of Regensburg, Universitätsstraße, Regensburg, Germany; 7 Department of Biology, Pennsylvania State University, 208 Mueller Laboratory, University Park, PA, USA; 8 Department of Fish, Wildlife and Conservation Biology, Colorado State University, Fort Collins, CO, USA; 9 Biology Department, Woods Hole Oceanographic Institution, Woods Hole, MA, USA; 10 Nicholas School of the Environment, Duke University, Durham, USA; 11 Department of Biology, University of Washington, Seattle, WA, USA; 12 Department of Evolution, Ecology and Organismal Biology, The Ohio State University, Columbus, OH, USA; 13 Department of Wildlife Ecology and Conservation, University of Florida, Gainesville, FL, USA; 14 CRS Forestry Commission, Calabar, Nigeria; 15 National Socio-Environmental Synthesis Center, 1 Park Place, Annapolis, MD, USA; 16 Naturschutz & Landschaftsökologie, Albert-Ludwigs-Universität Freiburg, Freiburg, Germany; 17 Department of Geography, Texas A&M University, College Station, TX, USA; 18 Centre for Ecology & Hydrology, Wallingford, UK; 19 Wildlife Biology Program, University of Montana, Missoula, MT, USA; 20 Department of Mathematics, University of Miami, 1365 Memorial Drive, Coral Gables, FL, USA; 21 University of California-Davis, Davis, CA, USA; 22 Department of Mathematics, Howard University, Washington, DC, USA; 23 Physics Department, California Polytechnic State University, San Luis Obispo, CA, USA; 24 Department of Biology, College of Charleston, Charleston, SC, USA; 25 Department of Ecology and Evolutionary Biology, University of Connecticut, Storrs, CT, USA; 26 Environmental and Plant Biology, Ohio University, Athens, OH, USA; 27 Department of Mathematics, University of Tennessee, Knoxville, TN, USA; 28 Department of Mathematics, Lafayette College, Easton, PA, USA

**Keywords:** dependency, directed dispersal, dispersal vectors, generalization, mutualism, seed dispersal effectiveness

## Abstract

Seed dispersal enables plants to reach hospitable germination sites and escape natural enemies. Understanding when and how much seed dispersal matters to plant fitness is critical for understanding plant population and community dynamics. At the same time, the complexity of factors that determine if a seed will be successfully dispersed and subsequently develop into a reproductive plant is daunting. Quantifying all factors that may influence seed dispersal effectiveness for any potential seed-vector relationship would require an unrealistically large amount of time, materials and financial resources. On the other hand, being able to make dispersal predictions is critical for predicting whether single species and entire ecosystems will be resilient to global change. Building on current frameworks, we here posit that seed dispersal ecology should adopt plant functional groups as analytical units to reduce this complexity to manageable levels. Functional groups can be used to distinguish, for their constituent species, whether it matters (i) if seeds are dispersed, (ii) into what context they are dispersed and (iii) what vectors disperse them. To avoid overgeneralization, we propose that the utility of these functional groups may be assessed by generating predictions based on the groups and then testing those predictions against species-specific data. We suggest that data collection and analysis can then be guided by robust functional group definitions. Generalizing across similar species in this way could help us to better understand the population and community dynamics of plants and tackle the complexity of seed dispersal as well as its disruption.

## Teaser

Seed dispersal is critical to plant fitness and plant community dynamics. However, measuring and tracking all factors that influence seed dispersal effectiveness for any potential seed-disperser relationship would require an unrealistically large amount of time, materials and financial resources. Building on current frameworks, we suggest that seed dispersal ecology quantify seed dispersal at the scale of plant functional groups, in order to reduce complexity to manageable levels. Based on functional group classifications, plant species can be distinguished by their level of dependence on seed dispersal and its mechanisms.

## Introduction: Seed Dispersal Is Fundamental to Populations and Communities, Yet Complex

Plants rely on dispersal vectors—for example, animals, wind and water—to move across the landscape. We focus here on the dispersal of seeds, although many of the arguments we make could be generalized to other forms of dispersal. Dispersal occurs when a seed is moved from its origin and deposited elsewhere (Schupp *et al.* 2010). Through dispersal, plants may experience reduced exposure to competition, predation and parasitism (Janzen 1970; Connell 1971; Howe and Miriti 2004); colonize open habitats after disturbance (Wunderle 1997; Puerta-Piñero *et al.* 2013); reach potential suitable microsites in otherwise unsuitable landscapes (Wenny 2001); track climate fluctuations and environmental change (Corlett and Westcott 2013); and contribute to gene flow within and between populations (Bacles *et al.* 2006). As a result of these processes, seed dispersal is a fundamental driver of the diversity, structure, composition and spatial arrangement of plant communities. Seed dispersal ecology thus elucidates mechanisms of species coexistence, implications of species extinctions and impacts of global environmental change.

It is evident that a quantitative understanding of dispersal is key for predicting how environmental changes, and consequent changes in dispersal vectors, will impact plant populations and communities. Operationalizing this goal and moving seed dispersal ecology towards a predictive science, however, requires confronting a wide array of interacting factors and stochastic elements (Robledo-Arnuncio *et al.* 2014). Here, we discuss how a functional group approach may help simplify the complexity of seed dispersal ecology and boost our predictive capacity.

Functional group frameworks, in which species are categorized by ecological functions and the resulting groups treated as analytical units, have helped researchers confront complexity in other ecological subdisciplines and have been tentatively explored in seed dispersal (e.g. Dennis and Westcott 2006; Brodie *et al.* 2009b; Bastazini *et al.* 2017). However, they have not yet been developed sufficiently to link empirical patterns of seed dispersal with theoretical predictions. In this Viewpoint, we discuss the complexity of seed dispersal and the need to reach generalities about it. We propose that to better understand the importance of seed dispersal in plant populations and communities, it would be useful to identify functional groups that distinguish plant species based on (i) how much it matters if their seeds are dispersed at all, (ii) how much it matters into what ecological context they are dispersed and (iii) how much it matters by what vector they are dispersed. We list such functional groups and discuss their potential value in achieving general insights. We close by considering key knowledge gaps that this proposed functional group approach may address.

### The complexity of seed dispersal

**Figure 1.**
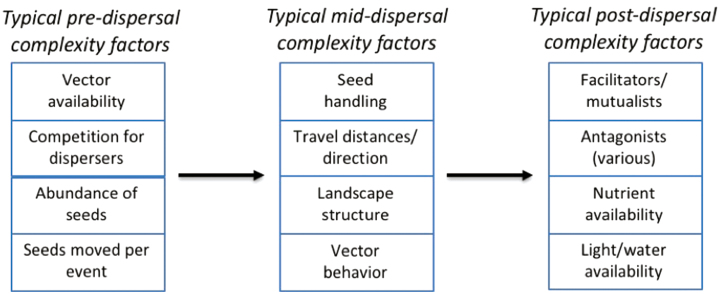
Seed dispersal exemplifies ecological complexity. Survival to adulthood and the fitness of individual adults are influenced by pre-, mid- and post-dispersal variables including the availability of abiotic and biotic vectors; the behaviours, preferences, morphology and physiology of dispersers; the spatio-temporal heterogeneity in seed deposition locations; and the probability of encountering other mutualists, facilitators, predators, pathogens and competitors following dispersal.

Due to their complexity, seed dispersal processes are difficult to quantify empirically (Fig. 1). Since the quantification of these processes forms the basis for understanding plant population and community dynamics, methods to reduce this complexity are essential. Both biotic and abiotic dispersal vectors can influence which seeds are dispersed, the risks and costs of dispersal, the spatial direction and distance that seeds travel, the probability that seeds will encounter specific microhabitats and the probability of seed aggregation (Howe and Miriti 2004; Côrtes and Uriarte 2013; Morales *et al.* 2013). For seeds transported by abiotic vectors, wind and water speeds and turbulence determine the distance and direction of seed movement (Katul *et al.* 2005; Nathan *et al.* 2011): not only are these factors intrinsically variable, but that variation interacts with the physical structure of the environment and the size and shape of the seed. In biotic dispersal, the set of disperser animals interacting with a seed may dictate its survival, growth and eventual reproduction (García and Martínez 2012). Dispersal vectors vary in their interactions with landscape structure, implying that the mechanism of dispersal may dictate the composition and arrangement of a plant community (Metzger 2000; Albrecht *et al.* 2012; Effiom *et al.* 2013; Razafindratsima and Dunham 2016; Chen *et al.* 2017). We largely focus on biotic seed dispersal because the behaviours and physiology of biotic dispersers amplify the complexity of seed dispersal. Seed handling, for example, can affect the condition of the seed and change the likelihood of germination and subsequent survival and growth after seed deposition (Ladley and Kelly 1996; Traveset and Verdú 2002; Fricke *et al.* 2013). Some plant species exhibit extreme specialization in microhabitats and require dispersers to move seeds to these locations (e.g. desert mistletoe requires dispersal to the branches of a very limited range of host trees; Aukema 2004). The preferences and physiology of dispersers may influence the direction and distance of seed dispersal (Beckman and Rogers 2013) (Fig. 1). Stochastic events may include rare, long-distance dispersal events, which are difficult to observe and measure but can be critical for colonization of new geographic regions and provide connectivity among habitat patches across a landscape (Muller-Landau *et al.* 2003; Jordano *et al.* 2007; Shea 2007; Auffret *et al.* 2017). Behavioral aspects of biotic dispersers, such as local aggregation, social organization, mating system, competition and territoriality, can influence both spatial and temporal dispersal of seeds, with potential ramifications for seed aggregation and competition between seeds (reviewed in Karubian and Durães 2009). A given disperser may also disperse seeds of certain shapes or sizes, depending on disperser body or gape sizes (McConkey and Drake 2006; Muñoz *et al.* 2017). An extensive literature has explored the dispersal syndromes, or seed and fruit traits (e.g. size, shape, colour, chemistry, dormancy) that appear predictive of the primary dispersers of a given plant species, with investigation into the roles of co-evolution, secondary dispersal and specialization (e.g. Vander Wall and Beck 2012; Howe 2016). Below, we briefly touch on the importance of dispersal syndromes as a form of functional grouping that categorizes dispersal adaptations. However, our proposed framework focuses instead on functional group delineations that distinguish the importance of dispersal in plant populations and communities.

Where and when a seed is deposited are clearly influenced by many sources of variability (Robledo-Arnuncio *et al.* 2014). Additionally, the spatial pattern of seed deposition on the landscape can then influence subsequent interspecific interactions (e.g. pollination, mycorrhizal associations, competition, predation, herbivory). Such interactions are important to the fitness of the newly established plant and determine the likelihood of survival and growth, access to limiting resources, the likelihood of mortality due to natural enemies and the probability of successful reproduction (Beckman and Rogers 2013). As a result of these interactions, the resulting plant community may more or less closely reflect the initial template established by seed deposition.

### Seeking predictive capacity in light of global change: adapting current frameworks for functional groups

As established above, the plant community in a given location is constrained by the template established by seed deposition, but the post-deposition interactions within the seedscape (i.e. the full environmental context into which the seed is dispersed) determine which subset of those seeds succeeds. Empirically quantifying all relevant pre- and post-dispersal variables is a complex task for even one plant-disperser pair, and impossible for the thousands of species pairs that participate in seed dispersal mutualisms worldwide (Howe and Smallwood 1982; Aslan *et al.* 2013; Beckman and Rogers 2013). Nevertheless, without an attempt to understand these processes, their variability and the drivers of that variability, our understanding of system dynamics is hamstrung.

The Seed Dispersal Effectiveness (SDE) framework is a comprehensive framework to summarize the full suite of variables affecting the dispersal service provided to any particular plant species by any particular vector (Schupp *et al.* 2010). The SDE framework summarizes the contribution of each dispersal vector (whether biotic or abiotic) to the production of new adult plants by evaluating variables influencing the *quantity* of seeds dispersed and the *quality* of the seed dispersal event. Quantity metrics within SDE include, for example, the frequency of visits from the disperser to the plant species and the number of seeds dispersed per visit. Quality metrics include, for example, the condition of the deposited seed (which depends, e.g., on an animal’s seed-handling behaviour) or a disperser’s movement patterns combined with measures of habitat quality across the landscape (Schupp *et al.* 2010). The SDE framework examines the complexity of variation within and among seeds, dispersers and other interactors determining the likelihood that a seed grows into a seedling (and, ultimately, reproductive adult), given a specific vector moving that seed to a specific location (e.g. Alvarez-Buylla and Martinez-Ramos 1990; Godinez-Alvarez and Jordano 2007; Escribano-Avila 2014; Rey and Alcántara 2014; Rother *et al.* 2016).

A full utilization of the SDE framework involves quantifying the effectiveness of dispersal for interacting pairs of seed and disperser species, taking into account pre-, mid- and post-dispersal factors that might affect seed survival and germination and the growth and fecundity of the resulting plant (e.g. Fig. 1). However, parameterization of SDE requires immense investment of empirical resources and includes up to 15 different measurable quantities for a given seed-disperser pair (Schupp *et al.* 2010). In one study, plant species were dispersed by an average of just over seven different disperser species (Aslan *et al.* 2013); parameterization of SDE for such a plant would therefore require a minimum of 7 × 15 = 105 separately measured parameters—a degree of complexity that would exhaust the resources of most scientific endeavours. Nevertheless, SDE has guided impressive efforts to measure subsets of these parameters, generating important insights. For example, McConkey *et al.* (2014) measured disperser effectiveness as a combination of the percent of monitored fruit dispersed by each disperser species combined with the distance of dispersal and survival of seedlings at each distance. Nogales *et al.* (2017) compared the number of seeds dispersed and effect of gut treatment by reptile vs. bird frugivores in the Galápagos. González-Castro *et al.* (2015) combined the number of seeds dispersed with condition of seeds after dispersal and seedling emergence/survival probabilities to compare SDE for birds and lizards. As these studies illustrate, different dispersers contribute in different ways to the template constraining an eventual plant community. To understand these roles across many more sites and for many more species, we require approaches that build off the SDE framework while simplifying the complexity inherent in biologically diverse systems.

To achieve this goal, we propose using plant functional groups in place of individual species in the SDE framework (Table 1). Functional groups are employed in many fields of ecology and have proven to be useful (e.g. functional group classifications yielded insights into plant species responses to climate change in Africa, Scheiter and Higgins 2009; successional dynamics in a Costa Rican forest, Chazdon *et al.* 2010; and global vegetation patterns, Sato *et al.* 2007). By identifying relevant traits, functional group frameworks unite species sharing those traits under a common lens allowing generalization across diverse organisms. Because functional groups by definition describe the ecological functions present in a given site, functional group diversity has in some cases been found to predict whole-ecosystem function almost as well (or better than) species diversity (Díaz and Cabido 2001). At the same time, functional groups are conceptual constructs and thereby subject to the perspective of ecologists identifying traits they deem important to particular questions. Beginning with SDE allows us to anchor our functional group recommendations in a robust and established guiding comprehensive framework. Thus, the three broad categories of functional groups described below have been selected to distil the comprehensive SDE framework into straightforward conceptual bins. We acknowledge that other approaches to simplifying matters are possible, perhaps based on different criteria, or on different quantifications of the same criteria. However, as we demonstrate here, considerable insights can be obtained with our approach. It is also important to acknowledge that, compared with a species-level SDE analysis, a functional group-level SDE analysis carries a risk of overgeneralization, whereby meaningful sources of variation are dismissed due to limited understanding.

**Table 1.** Functional groups relevant to the importance of seed dispersal for seed survival and thereby plant population and community dynamics. We propose that researchers and conservation planners determine whether target plant species belong to functional groups for which dispersal disruption is likely to significantly decrease fitness vs. have only minor effects on fitness. These groups are categorized based on how much it matters whether a seed is dispersed (shown in red; groups 1–3); how much it matters where or when dispersal occurs (shown in blue; groups 4–14); and how much it matters what vector disperses the seed (shown in green; groups 15–17). Applying vulnerability assessments and SDE calculations at the level of these functional groups may enable us to achieve a predictive understanding of seed dispersal ecology in the face of combined global change and complexity. *For species exhibiting a measurable fitness boost from dispersal, seed size may dictate which abiotic or biotic vectors are effective.

Functional group category	Characteristics of functional groups likely significantly affected by seed dispersal disruption	Characteristics of functional groups likely less affected by seed dispersal disruption	Sample references
Groups for which plant fitness is affected by whether seeds are dispersed.	High colonization ability	High competitive ability	Coomes and Grubb (2003)
	Long-distance dispersal adaptations	Local dispersal adaptations	Muller-Landau *et al.* (2003)
	Density-dependent survival	Density-independent survival	Rey and Alcántara (2014)
Groups for which plant fitness is affected by where or when dispersal occurs.	Thin/vulnerable seed coats	Thick/hard/spiky seed coats	Notman and Gorchov (2001)
	Shade-intolerant	Shade-tolerant	Alvarez-Clare and Kitajima (2007)
	Fire-intolerant	Fire-tolerant	Wenny (2001)
	Self-incompatible	Self-compatible	Bond (1994)
	Reproduction by seed only	Reproduces asexually	Bond (1994)
	Intolerant of low nutrients	Tolerant of low nutrients	Wenny (2001)
	Low phenotypic plasticity	High phenotypic plasticity	Goh *et al.* (2013)
	Metapopulation-dependent	Continuous population distribution	Bohrer *et al.* (2005)
	Negative distance-dependent mortality	No negative distance-dependent mortality	Beckman *et al.* (2012)
	Inability to seed bank	Seed banking	Gutterman (2000)
	Seasonal dispersal	Low dispersal seasonality	Ruggera *et al.* (2015)
Groups for which plant fitness is affected by the vector of dispersal	Seed size*	Seed size*	Tamme *et al.* (2014)
	Intraspecific competitor/non-facilitator	Intraspecific facilitator	Martorell and Freckleton (2014)
	Seed coat with germination inhibitors	No seed coat germination inhibitors	Traveset and Verdú (2002)

Previous uses of functional groups in seed dispersal ecology have been narrow in scope (focusing on single systems or a small number of focal functions) but are indicative of the usefulness of this approach. For example, Dennis and Westcott (2006) distilled 26 detailed measurements of seed disperser traits into 10 trait dimensions. They used these dimensions to identify 15 functional groups in a suite of 65 Australian seed disperser species; their mathematical approach could be more broadly applied to reduce complexity in other systems (Dennis and Westcott 2006). Rodríguez-Rodríguez *et al.* (2017) categorized plant–animal interactions into typologies and evaluated links between these typologies and plant fitness. Zamora (2000) explored how the consistency of fitness benefits offered by seed dispersal across systems and groups of species permits generalization within functional groups. A key benefit of a functional group approach is that it could provide an understanding of the functions that may be lost when extinctions occur (Blondel 2003; Bastazini *et al.* 2017). This conservation-oriented conceptual application was highlighted by Schleuning *et al.* (2014) in their call for more work examining the linkage between trait-based approaches such as functional group delineation and structural approaches such as network analysis (Ruggera *et al.* 2015). Functional groups can be used to predict the role of suites of species in an ecosystem and the response of those species to drivers of global change.

## Meaningful Functional Groups in Seed Dispersal Ecology

We define plant functional groups based on traits influencing the importance of seed dispersal for plants (Table 1). Our proposed functional groups categorize plants based on: (i) how important it is to plant recruitment if seeds are dispersed at all, (ii) how important the location and timing of seed deposition are and (iii) how much vector identity matters. These groups thus define important points in the dispersal process at which seed fate may be influenced, with a focus on the fitness benefits derived from dispersal events. Applying SDE to these functional groups will enable researchers to predict how populations of the species within a group will be affected by total or partial dispersal disruption, changes in phenology or habitat conditions, or entry of non-native species into dispersal networks. A given plant species may display traits that make them likely vulnerable to dispersal disruption based on one functional group category and less vulnerable based on another; in such a case, these categories will help to pinpoint sources of such vulnerability.

### Functional groups distinguishing how much it matters if a seed is dispersed at all

Species may be categorized based on the importance of dispersal for their survival and reproduction. Well-established frameworks examining fitness benefits that may be derived from seed dispersal can guide functional group determination in this arena, since functional groups can be defined by traits linked to such fitness benefits. The *escape hypothesis* states that seeds will experience fitness boosts as a result of removal from the neighbourhood of natural enemies, including pathogens, parasites, herbivores and competitors (Howe and Smallwood 1982; Howe and Miriti 2004). Escape from beneath the canopy of a parent tree reduces the chances of pathogens and herbivores finding a seed (Janzen 1970; Connell 1971), as well as the chance that a seed will be deposited immediately adjacent to a close relative and thus compete for necessary resources. A logical extension of this hypothesis suggests that species can be assembled into functional groups by traits indicating dependence upon such escape (i.e. susceptible to infection or herbivory; exhibiting negative density-dependence in survival and growth) vs. those less dependent upon escape (i.e. exhibiting thick seed coats or other protections against infection and herbivory; exhibiting low negative density-dependence). As an example, in a study of olive (*Olea europaea*) regeneration in human-altered vs. unaltered landscapes in Spain, proximity to maternal trees was associated with elevated seedling mortality; *O. europaea* thus appears to occupy a functional group characterized by escape dependence and negative density-dependence (Rey and Alcántara 2014) (Table 1). Similarly, fungal pathogens led to strong density-dependent mortality in *Pleradenophora longicuspis* in Belize, evidence that functional group categorization based on density-dependence is appropriate for this species (Bagchi *et al.* 2010). By contrast, species with low density-dependence, and thus likely to be classified into functional groups with reduced dispersal-dependence, include a suite of common species in a Panamanian rainforest, where density-dependence varies considerably among tree species (Comita *et al.* 2010). Species with greater seed mass exhibited reduced negative density-dependence on Barro Colorado Island (Lebrija-Trejos *et al.* 2016).

Previous species-specific studies have examined density-dependent damage and mortality in seeds and seedlings encountering abundant herbivores, pathogens and predators in close proximity to parent trees (the Janzen–Connell effect) (e.g. Petermann *et al.* 2008; Bagchi *et al.* 2010; Liu *et al.* 2012). Study results have been mixed, but largely show increased success of seeds and seedlings after removal from the parent, with various explanatory mechanisms (e.g. Thomas 1990; Blundell and Peart 1998; Packer and Clay 2000; Petermann *et al.* 2008; Bagchi *et al.* 2014). These studies suggest that assigning seeds to functional groups based on whether they exhibit negative density-dependence is important. For those groups that do exhibit such density-dependence (Table 1), loss of dispersers may be expected to affect plant fitness significantly. Assigning plants to functional groups may in some cases be possible through observational studies, generalizing from what we know about similar species, and in other cases may require experimental assessments—which are still far less extensive than a traditional species-specific SDE assessment (Table 1).

Interspecific interactions can affect parameters of matrix population models, enabling their effect on fitness to be examined using elasticity and sensitivity analyses (McGraw and Caswell 1996; Horvitz *et al.* 1997; Benton and Grant 1999; Mills *et al.* 1999; Carslake *et al.* 2009; Jongejans *et al.* 2011). The effect of seed dispersal failure can be explored via elasticity analyses simulating loss of dispersers and resulting failure to escape from natural enemies or encounter recruitment sites (Howe and Miriti 2004; Brodie *et al.* 2009a; Rodríguez-Pérez and Traveset 2012; Traveset *et al.* 2012; Caughlin *et al.* 2015; Pérez-Méndez *et al.* 2015). Applying such analyses to functional groups that enable generalization beyond a few carefully measured surrogates to other species within a group might greatly expand the predictive capacity of such analyses across systems.

Other fitness benefits of seed dispersal may arise from *colonization* of unpredictable and newly available germination sites and *directed dispersal* to hospitable microsites located within a non-hospitable matrix (Wenny 2001; Howe and Miriti 2004). Dispersal is likely to matter most to functional groups of species with specialized spatio-temporal germination and growth site requirements or low competitive ability and thus high dependence on vacant establishment sites. Identification of such species may be informed by competition/colonization trade-off theory, which predicts that species exhibit a trade-off between dispersal ability and competitive ability (for example, plant species may trade off the production of a few large, well-provisioned seeds for the production of many smaller seeds) (e.g. Bolker and Pacala 1999; Dalling and Hubbell 2002; but see Coomes and Grubb 2003). Similarly, theoretical ecologists have investigated when long-distance dispersal vs. local dispersal is evolutionarily advantageous, given the fitness advantages of colonizing new sites and the lower probability of finding habitats sharing specific characteristics at greater distances from one another (Snyder and Chesson 2003; Snyder 2011).

### Functional groups distinguishing how much it matters where or when a seed is dispersed

A large fraction of the ‘quality’ element of the SDE framework centres on where and when a seed is dispersed. Fundamentally, this will dictate which abiotic and biotic resources and threats are encountered by the seed and subsequent plant (Schupp *et al.* 2010; Beckman and Rogers 2013). Abiotic resources may include nutrients, moisture, space and light. Abiotic threats could include drought, nutrient deficiencies, frost and fire. On the biotic side, resources could include mutualists such as soil mycorrhizae, pollinators, seed dispersers and facilitators, and threats could include herbivores, competitors, predators and pathogens. Functional groups that categorize species by whether deposition setting matters to a seed may include (i) groups of plants that are particularly susceptible to abiotic stressors/disturbances or natural enemies (e.g. plants with low competitive ability or thin seed coats) vs. (ii) those tolerant of threats (e.g. shade-tolerant, fire-tolerant, drought-tolerant, etc.). Other relevant functional groups would include species dependent on mutualists or facilitators (Calvo and Horvitz 1990; Onguene and Kuyper 2002; Hoehn *et al.* 2008; Teste *et al.* 2009), frost-intolerant species that require nurse plants, species dependent on forest gaps to escape shading and species that require a narrow range of soil nutrient content.

Plant species with plastic phenotypes may be relatively generalized with regard to their interspecific interaction requirements, suggesting that functional groups defined by plasticity may be appropriate. Plasticity may influence dependence upon certain abiotic conditions or interspecific interactions. For example, mycorrhizal associations could provide critical assistance to plant individuals with delicate or small root systems, but individuals with plastic growth (e.g. those able to divert resources towards robust root growth as required) might be less affected by an absence of root symbionts (Valladares *et al.* 2007; Goh *et al.* 2013).

Dispersal also matters for plants living in habitats that are temporally or spatially variable. Important functional groups include those with specific habitat requirements that are spatially heterogeneous (e.g. species dependent on metapopulation processes for persistence; Bohrer *et al.* 2005) vs. general habitat requirements that are widespread and homogeneous. For example, in a human-disturbed, patchy landscape, affinity of dispersers for seedling habitat leads to increased germination of the relic Chinese yew (*Taxus chinensis*), indicating that directed dispersal matters for this endangered plant species (Li *et al.* 2016) (Table 1). In another example, seeds of the shrub *Daphne rodriguezii* dispersed to sites below nurse plants exhibit higher seedling survival (Rodríguez-Pérez and Traveset 2010) (Table 1). Lower location specificity can be found in, for example, shade-tolerant species that exhibit physical defence mechanisms and are thus able to survive and reproduce in conditions of high competition; eight such species were studied in Panama and their physical traits documented (Alvarez-Clare and Kitajima 2007). This indicates that material characteristics can be identified to classify such species into functional groups with less dependence on dispersal (Table 1). Some functional groups may be affected by positive or negative distance- or density-dependent mortality (e.g. if seeds must be dispersed in clumps to germinate and grow; Beckman *et al.* 2012), or may require rare micro-conditions (Pufal and Garnock-Jones 2010). Temporally, some species can protect themselves against poor dispersal years by living many years as adults or remaining viable in a seed bank for a long time (e.g. Gutterman 2000). In other cases, the timing of dispersal interacts with characteristics that determine habitat quality (e.g. ephemeral environmental conditions or seasonally migratory dispersers) (Ruggera *et al.* 2015). Timing can matter on the plant side, too: in a study of *Pistacia lentiscus* dispersal in Spain, seed viability was found to vary during the fruiting season, such that dispersers interacting with the species when viability is high were more effective than those handling fruits at other times (González-Varo *et al.* 2018).

In theory, the functional groups most dependent upon dispersal include species in patchy habitats, those with strong density-/distance-dependent mortality, those lacking the ability to maintain a seed bank and those with specific requirements for the timing and location of the dispersal event (Table 1).

### Functional groups enabling us to distinguish how much the identity of the dispersal vector matters

Dispersal syndromes are used to categorize plants by the type of vector known or assumed to best disperse their seeds. Syndromes are the most common functional group classifications used in seed dispersal ecology. Illustrating the potential value of generalization across similar dispersers, Tamme *et al.* (2014) successfully used plant traits to predict dispersal distances for over 500 species. Dispersal distances could then be related to dispersal syndromes, growth form and other plant traits, such as plant height and seed size (Thomson *et al.* 2011; Tamme *et al.* 2014). Previous studies have reported an interaction between seed size and dispersal vector size, as seed size sets a lower limit on the type and size of dispersal vector that can lift (e.g. wind) or ingest (e.g. animals) the seed (Wheelwright 1985; Ganeshaiah and Shaanker 1991; McConkey and Drake 2002). In some cases, dispersal syndromes explain some variation in dispersal distances and can be used to predict dispersal distances (Tamme *et al.* 2014), but the variation within dispersal syndromes can be very high (Clark *et al.* 2005; Muller-Landau *et al.* 2008). Dispersal syndromes tend to be broad categories (e.g. large mammal vs. small mammal vs. wind). Even within these categories, species may be dispersed by a diversity of vectors, and in some cases secondary dispersal is performed by an altogether different class of vector than primary dispersal (Böhning-Gaese *et al.* 1999; Vander Wall and Beck 2012). Whether the identity of the vector matters to the eventual success of the seed is an important component of understanding the role of dispersal in eventual plant population and community dynamics.

In spite of these successful attempts to achieve general insights, there are certain risks associated with generalizing across vectors (or dispersers). Identifying a dispersal syndrome may suggest that a broad category of vector is the likely disperser, but such categories could include many potential disperser species varying in effectiveness (Jordano *et al.* 2007; Howe 2016). Thus, dispersal syndromes are not sufficient to predict the effects of losing certain vectors. Nor do dispersal syndromes give us information on the likelihood of being dispersed by a ‘non-standard’ dispersal vector—that is, a vector other than the most common vector or vectors interacting with a particular plant—which might be more influential than ‘standard’ vectors in long-dispersal events (Higgins *et al.* 2003; Jordano *et al.* 2007) and therefore exert larger effects on plant populations (Kot *et al.* 1996; Neubert and Caswell 2000). As an important lesson for dispersal ecology, the concept of syndromes has faced substantial criticism in pollination ecology (Ollerton *et al.* 2009). Careful empirical study has demonstrated that in most cases both plants and pollinators are much more opportunistic and interact with a much broader suite of partners than morphological pollination syndromes would suggest (Waser *et al.* 1996; Fenster *et al.* 2004; Ollerton *et al.* 2009; Waser *et al.* 2018). If syndromes are similarly uninformative in dispersal, this carries implications for conservation and management, since incorrect generalization stemming from syndromes could lead to fallacious assumptions about the redundancy of dispersers within interaction networks and, consequently, about restoration and conservation needs (Howe 2016).

Although the use of dispersal syndromes *per se* thus carries a risk of drawing conclusions at too crude a scale, straightforward functional groups founded on seed morphology and physiology (e.g. determined by seed size and shape) may dictate potential disperser suites and indicate how important different dispersal vectors may be, relative to one another, for a given plant species (Table 1). Identifying plant species at risk from dispersal disruption (e.g. McConkey *et al.* 2018) may be possible when the importance of vector identity is understood. Vectors may differ in the number of seeds dispersed, the condition of dispersed seeds, dispersal distances and dispersal spatial arrangements. Wind, for example, is most likely to move seeds that are small in mass (Shea 2007; Nathan *et al.* 2011). Disperser animals with large gape sizes are more likely than small dispersers to disperse greater numbers of larger seeds over longer distances (Cox *et al.* 1991). Since large dispersers with low reproductive rates are often most threatened by direct human exploitation coupled with low reproductive rates (Farwig and Berens 2012), the plant functional group that includes large-seeded species is of particular interest in seed dispersal conservation. Losses of key large dispersers can threaten plant species and functional group diversity in seed dispersal networks (Donoso *et al.* 2017). Dispersers with specialized habitat requirements may aggregate seeds by returning frequently to a limited number of sites (Howe 1989). Because different vectors may provide dispersal services in different ways, plant species may experience complementary dispersal services from them, with a greater diversity of vectors maximizing the success of a plant (Levin *et al.* 2003; Jordano *et al.* 2007; Bueno *et al.* 2013; Escribano-Avila *et al.* 2014; González-Varo *et al.* 2017). Plant functional groups of interest when determining whether a specific vector is important include groups defined by seed size, seed coat thickness (e.g. groups of species with thick coats requiring substantial gut treatment for germination), presence of germination inhibitors, and intraspecific facilitation or positive density-dependence.

Exemplifying the importance of this functional group delineation, different behaviours of large mammal dispersers resulted in differential contributions to dispersal of the large-seeded *Platymitra macrocarpa* in Thailand, with some species dispersing higher quantities of seeds with poor survival outcomes and others dispersing fewer seeds with greater success per seed (McConkey *et al.* 2018). In this case, dispersers contributed differentially to the dispersal of the plant but overall plant regeneration was poor, leading researchers to speculate that there may be important dispersers that are missing or rare (McConkey *et al.* 2018). In that context, the large seed size of the plant suggests that the identity of the dispersal vector in this example is important according to the functional groups we propose (Table 1) (McConkey *et al.* 2018). By contrast, SDE of a suite of bird species was studied for two *Miconia* species in Brazil (Santos *et al.* 2017). Although the birds varied in the quantity of seeds they dispersed, they did not vary in quality of dispersal (Santos *et al.* 2017). *Miconia* species with their small seeds and large disperser suites (e.g. Levey and Byrne 1993) therefore appear to fall into a proposed functional group for which vector identity is less important (Table 1).

Dispersal vector identity has been shown to affect population growth rates for some but not all of the few vertebrate-dispersed plant species that have been studied (e.g. Godinez-Alvarez and Jordano 2007; Brodie *et al.* 2009b; Loayza and Knight 2010). However, the importance of different vectors is unknown for most plant species, and that lack of clarity hampers our ability to predict the outcomes of changes in vectors. Predictions are better-developed for ballistic- and wind-mediated dispersal than for animal-mediated dispersal (Skarpaas and Shea 2007; Nathan *et al.* 2011; Bullock *et al.* 2012), in large part because of the complexity of animal behaviour and movement and the diffuse nature of most seed dispersal systems, wherein multiple animals disperse any given plant (Shea 2007). Even when detailed information about the role of specific vectors has been obtained for a given plant species, studies are often narrow in spatial and temporal extent and thus context-dependent (i.e. information is specific to a particular time and place, given a particular disturbance history), and the importance of individual vectors may change under different contexts.

## Using Functional Groups to Close Our Knowledge Gaps

The use of functional groups defined by dispersal-related traits can reduce the amount of data needed to parameterize models (Mokany *et al.* 2014). The digital availability of trait data is increasing (e.g. via publicly accessible databases such as TRY; Kattge *et al.* 2011) but continued empirical research is needed to relate those data to dispersal processes. Even so, certain functional groups can now be defined and used to distinguish species that are relatively more or less strongly dispersal-dependent (Table 1). If a particular plant species belongs to a group for which fitness is strongly linked to dispersal (Table 1, column 2), we can predict that this species is likely to be vulnerable in the face of dispersal disruption, based strictly on functional group.

While functional groups may enable us to generalize across full plant communities, overgeneralization could cause us to lose sight of meaningful sources of variation. It is thus necessary to test the value of functional group approaches in seed dispersal ecology by generating predictions based on them and then testing those predictions against species-specific data. As functional groups ‘pass’ these tests, our ability to generalize by constructing our models and predictions around functional groups will help us manage the broad variability and context-dependence that can characterize seed dispersal events (Robledo-Arnuncio *et al.* 2014).

Compiling large amounts of data from multiple systems to test functional group approaches can be resource-intensive, although increasingly global databases are becoming available and serving as common data hubs (e.g. COMPADRE Plant Matrix Database and COMADRE Animal Matrix Database; Max Planck Institute for Demographic Research (Germany); available at www.compadre-db.org). One promising approach is to standardize data collection by many research groups working across many systems (e.g. NutNet; Borer *et al.* 2014). If groups collect the same data across systems, those data can be introduced to a common modelling platform to explore patterns that hold across systems. By assembling data sets in this way, the costs are spread across research groups, and data collection can be useful even when sample sizes within a specific system are limited. As an example, standardized data collection across systems has enabled researchers to identify consistent patterns of grassland responses to land use change (Garnier *et al.* 2007). We thus recommend that research teams join forces to collect standardized data exploring varying effectiveness of dispersal by different agents, the role of spatio-temporal dynamics and the influence of interspecific interactions pre-, mid- and post-dispersal. Once data are collected in many systems, it will be necessary to bring them together to make them available for broad analysis. Depositing seed dispersal data into public-access repositories is therefore important. Useful repositories include Dryad (http://datadryad.org) and KNB (http://knb.ecoinformatics.org).

To grow our understanding of seed dispersal ecology and to predict the likely effects of environmental changes, we advocate a new focus on seed dispersal functional groups. The plant functional groups described here are defined based on plant dependence on seed dispersal for plant population persistence and the likelihood of experiencing meaningful dispersal disruption. Analysis at a functional group level generalizes across species and systems and may enable us to more effectively assess the role of seed dispersal in the shaping of plant populations and communities. We invite the ecological community to join us in this effort.

## Conflict of interest

None declared.

## Sources of funding

Ideas for this manuscript initiated during the Seed Dispersal Workshop held in May 2016 at the Socio-Environmental Synthesis Center in Annapolis, MD and supported by the US National Science Foundation Grant DEB-1548194 to N.G.B. and the National Socio‐Environmental Synthesis Center under the US National Science Foundation Grant DBI‐1052875. D.Z. received funding from the Swiss National Science Foundation (SNF, grant: PZ00P3_168136/1) and from the German Science Foundation (DFG, grant: ZU 361/1- 1).

## Contributions by the authors

C.A. led the development of the concepts, writing, and revising of the manuscript with input from N.G.B. and H.S.R. All authors contributed to the development of concepts and are listed in order of contribution and alphabetical order within each level of contribution.
